# A conversation with Alejandro Hoberman, MD, Executive Vice Chair of Pediatrics, Vice Chair of Clinical Research, Division Director, General Academic Pediatrics, and Distinguished Professor of Pediatrics and Clinical and Translational Science, and Jack L. Paradise, MD Endowed Professor of Pediatric Research; President, UPMC Children’s Community Pediatrics

**DOI:** 10.1017/cts.2022.502

**Published:** 2023-01-03

**Authors:** Kathy Siranosian

**Affiliations:** Clinical Research Forum, Washington, DC, USA

## Top 10 Clinical Research Achievement Awards Q & A

This article is part of a series of interviews with recipients of Clinical Research Forum’s Top 10 Clinical Research Achievement Awards. This article is with Alejandro Hoberman, MD, Executive Vice Chair of Pediatrics, Vice Chair of Clinical Research, Division Director, General Academic Pediatrics, and Distinguished Professor of Pediatrics and Clinical and Translational Science, Jack L. Paradise, MD Endowed Professor of Pediatric Research, President, UPMC Children’s Community Pediatrics. Dr. Hoberman and his colleagues studied whether tympanostomy tube placement, as compared with medical management, results in a greater reduction in the children’s rate of recurrence of ear infections. Dr. Hoberman received a 2022 Top 10 Clinical Research Achievement Award for *Tympanostomy Tubes or Medical Management for Recurrent Acute Otitis Media* [1]. *The interview has been edited for length and clarity.*

## How Did You Get Started in Clinical Research? Was There a Moment When You Knew You Were Hooked?

After finishing medical school at the National University of Buenos Aires, I did my residency at Children’s Hospital in Buenos Aires. During that time, when I was reading research about the most common pediatric infections – ear, sinus, throat – I noticed that many articles were coming from the same team, from Dr. Jack Paradise and Dr. Charles Bluestone at Children’s Hospital Pittsburgh. I was trained in pediatric care, and self-trained in pneumatic otoscopy, which is puffing a little air into the ear canal to move the ear drum and see if there is fluid behind it, but because I wanted to know more, I applied for fellowship at Children’s Hospital Pittsburgh. At that point, I wasn’t hooked on research. I was hooked on learning how to properly diagnose ear infections, and I was hooked on understanding what was making this team of general pediatricians, pediatric infectious diseases specialists, and ENT doctors so proactive about these frequently occurring pediatric problems. They were the ones answering questions that so clearly needed to be answered.

## That Fellowship Proved to Be the Turning Point?

Yes, I moved to the USA with my wife and my 9-month-old son. The co-directors of the fellowship program, Dr. Paradise and Dr. Ken Rogers, became my mentors and every day, we had a fellows’ course about the methodology of clinical research. It was working with this team that helped me realize that clinical research is what I wanted to do. I began to understand that I could help children not only by seeing them clinically but also by establishing standards and informing doctors through the evidence gathered by my research. After my fellowship, I was offered a faculty position at the University of Pittsburgh, where I now lead the division that Dr. Paradise led many years ago.

## You’ve Mentioned the Word “Team” a Few Times. How Does Teamwork Factor into Your Research?

It’s absolutely critical – clinical research is a team sport. Our work depends on the contributions of many different people. For example, in the paper that won the award, we needed to collaborate closely with ENT surgeons, nurses, nurse practitioners, research assistants, and many others who helped us follow the children over the trial duration (2 years) and analyze the data. Plus, of course, we couldn’t do this research without the children themselves and their parents. The children came in three or four times a year for regular checkups, and we also encouraged their parents to bring them in if they thought their child had an ear infection or was sick. They had my cell phone number, so they could call if they needed to. My number is 999-EARS so it’s easy to remember.

## Is It Routine for You to Give Parents Your Cell Phone Number?

Yes, it’s what I learned from my mentors and is an example of the enhanced care we provide to the parents who allow their children to participate in clinical trials. There are times now in my practice when I’m seeing the children of parents who used to be my patients when they were young – and even before them, there’s a long-standing history of quality pediatric care and research here. Parents of our patients know that they will be offered opportunities to participate in clinical trials, and they know that if they do, they are going to get high-quality care and direct access to us.

## What Are You Working on Now?

I am fascinated by the idea of helping pediatricians get better at diagnosing ear infections and I’m particularly excited about the use of artificial intelligence in diagnosing ear infections. In the 1990s, we developed an online curriculum called Enhancing Proficiency in Otitis Media (ePROM) to train residents about diagnosing ear infections. ePROM contained validated images, video, and expert content and was used extensively for many years. Now, I’m working on a different way to improve the diagnosis of ear infections – an advanced programming interface (API) that will allow pediatricians to upload a video image of the tympanic membrane and then receive a diagnosis of acute otitis media or not. We want to improve diagnosis, and we also want to move away from high-priced endoscopes by coupling this API to more scalable instruments, which could even be a mobile phone connected to an otoscope. That way, a clinician can easily visualize an image of the tympanic membrane on the large screen of a mobile device and through the API, get an answer about whether or not there is an ear infection. We are training the software with thousands of ear exams and are in the process of validating our results.

## It Sounds Like You Are Still Hooked on Learning How to Properly Diagnose Ear Infections?

Yes, I love what I do and I feel really energized about the projects I am working on and the patients I see. Next to the common cold, acute otitis media is the most frequently diagnosed illness in children in the USA. Improving the diagnosis of acute otitis media impacts hundreds of thousands of children – and their parents and caregivers – every year. What our team strives for, and what I learned from my mentors, is “to do the right thing.” That means diagnosing accurately and then selecting the right treatment. Our goal as researchers is to help clinicians by accruing the evidence that improves decisions.
